# Increased suicidal ideation in the COVID-19 pandemic: an employee cohort in Japan

**DOI:** 10.1192/bjo.2021.1035

**Published:** 2021-10-29

**Authors:** Natsu Sasaki, Reiko Kuroda, Kanami Tsuno, Kotaro Imamura, Norito Kawakami

**Affiliations:** Department of Mental Health, Graduate School of Medicine, The University of Tokyo, Japan; Division for Environment, Health, and Safety, The University of Tokyo, Japan; School of Health Innovation, Kanagawa University of Human Services, Japan; Department of Mental Health, Graduate School of Medicine, The University of Tokyo, Japan; Department of Mental Health, Graduate School of Medicine, The University of Tokyo, Japan

**Keywords:** Suicide, epidemiology, community mental health teams, primary care, psychological testing

## Abstract

**Objectives:**

This study investigated the change in suicidal ideation and its risk factors among employees. A longitudinal cohort study was conducted, starting with the baseline online survey in March 2020 (T1), followed by May (T2), and August (T3). The change in suicidal ideation from T2 to T3 and relevant factors associated with suicidal ideation at T3 were examined. Suicidal ideation significantly increased between May and August 2020 among females, younger (under 39 years old), highly educated population, and those without pre-existing mental health conditions. Factors significantly associated with suicidal ideation were younger age, suicidal ideation at T2, and with pre-existing mental health conditions. Loneliness at T2 showed a significant association with suicidal ideation, if adjusting those without pre-existing mental health conditions. National and community support is needed to target people who are likely to be left behind, such as young people and those with pre-existing mental health conditions, in the pandemic.

**Method:**

A longitudinal study was conducted with a cohort of full-time employees, starting with the baseline online survey in March 2020 (time point 1), followed by May (time point 2) and August (time point 3). The change in suicidal ideation from time point 2 to 3, and relevant factors associated with suicidal ideation at time point 3, were examined.

**Results:**

Suicidal ideation significantly increased between time points 2 and 3 among women, younger people (aged <39 years), those who were highly educated and those without pre-existing mental health conditions. Factors significantly associated with suicidal ideation were younger age, suicidal ideation at time point 2 and pre-existing mental health conditions. Loneliness at time point 2 showed a significant association with suicidal ideation when adjusting for those without pre-existing mental health conditions.

**Conclusions:**

National and community support is needed to target people who are likely to be left behind, such as young people, those with pre-existing mental health conditions and those experiencing loneliness, in the COVID-19 pandemic.

Suicide and suicidal ideation was a significant public health concern before the COVID-19 pandemic, but the pandemic has made it even more urgent.^1^ O'Connor et al reported that suicidal ideation increased among young people during lockdown (March to April 2020) in the UK.^2^ The results identified those aged 18–29 and 30–59 years at higher risk compared with those aged ≥60 years, suggesting that the pandemic is affecting the mental health of the working-age population more than the population aged ≥60 years. Another longitudinal survey conducted in the USA reported that suicidal ideation was more prevalent among employed than unemployed respondents.^3^ Suicidal ideation among the employed population should be an area of concern.

On 7 April 2020, the Japanese Government declared a state of emergency, which lasted until 25 May 2020. The Japanese Government and local governments did not impose a lockdown, but called on citizens to voluntarily refrain from non-essential and non-urgent outings and avoid the ‘three C's’ (confined spaces, crowded places and close contact). Although Japan's emergency declaration was much less restrictive than the lockdowns in Europe or the USA (comprising request-based measures for close or shortened business hours and stay-at-home requests instead of orders), poor mental health in the general public was reported.^4^ However, the effects on suicidal ideation and its risk factors, even after the conclusion of the state of emergency, have not been examined yet among the Japanese working population.

We conducted a longitudinal analysis on suicidal ideation by using the Employee Cohort Study in the COVID-19 pandemic in Japan (E-COCO-J),^5^ and investigated its risk factors.

## Method

Members of a pre-existing cohort were invited to take part. The cohort consisted of full-time employees recruited from community-dwelling people all over Japan by an internet research company in February 2019, stratified by gender and 10-year age groups (*N* = 4120). Through an invitation email from the internet research company, we further invited these respondents to participate in a baseline online survey of the E-COCO-J cohort. After completing an online baseline survey during 19–22 March 2020 (time point 1, *n* = 1448), the respondents, excluding the unemployed (*n* = 27), were invited to complete a survey during 22–26 May 2020 (time point 2) and 7–12 August 2020 (time point 3). The state of emergency was declared from 7 April to 25 May 2020. The survey time point is shown in Supplementary Fig. 1 available at https://doi.org/10.1192/bjo.2021.1035, along with the daily number of COVID-19 cases in Japan. At the peak of the first wave of the outbreak, Japan reported approximately 700 positive cases in a single day. Time points 1 and 2 are before and after the first wave of the COVID-19 pandemic, respectively, and time point 3 coincides with the peak of the second wave. Supplementary Fig. 2 shows the flow chart of participant recruitment. Suicidal ideation and loneliness in the past 30 days were measured by the items ‘I feel like I want to die’ and ‘I feel lonely’, respectively, at time points 2 and 3. The response option was scored with a four-point Likert scale: 1 (almost never), 2 (sometimes), 3 (often) and 4 (almost always). The variables were categorised into yes (2–4) and no (1). The McNemar test was used to assess differences in proportion of loneliness or suicide ideation for the total sample, and separately for groups classified based on gender, age, education, occupation type and pre-existing mental health conditions, between time points 2 and 3. Potentially relevant factors associated with suicidal ideation at time point 3 were analysed with the multiple logistic regression model (forced entry method), adjusted for gender, age, education, occupation type (measured in 2019), suicidal ideation and loneliness at time point 2, and pre-existing mental health conditions at time point 1 (current or past treatment for depression, anxiety or mood instability). This study was approved by the Research Ethics Committee of the University of Tokyo (approval number 10856-(2)(3)(4)(5)). Online informed consent was obtained from all participants, with full disclosure and explanation of this study's purpose and procedures. We explained that their participation was voluntary, and they could withdraw from the study for any reason simply by not completing the questionnaire.

## Results

The analytic sample consisted of currently employed respondents (*N* = 875) who responded to baseline and two follow-up surveys at time points 2 and 3. Participant characteristics are shown in Table 1. A comparison of respondents in the analytic sample (*N* = 875) and those who responded to time point 1 but dropped out at time point 2 or 3, or were excluded owing to unemployment (*n* = 573), showed that the analytic sample was more likely to be older (>40 years), male, employed in a managerial/non-manual occupation, have higher educational attainment and have no pre-existing mental health conditions (Supplementary Table 1).

**Table 1:** Characteristics of full-time employees during the COVID-19 pandemic in Japan (*N* = 875)

	*n* (%)	Mean (s.d.)
Gender		
Male	463 (52.9)	
Female	412 (47.1)	
Age		41.74 (10.4)
>40 years	492 (56.2)	
≤39 years	383 (43.8)	
Educational attainment		
Low (<16 years)	405 (46.3)	
High	470 (53.7)	
Occupation type		
Managerial/non-manual	648 (74.1)	
Manual	227 (25.9)	
Pre-existing mental health condition in March 2020 (time point 1)		
No	771 (88.1)	
Yes	104 (11.9)	

Suicidal ideation increased between time points 2 and 3 in the total sample (*P* = 0.008), as did loneliness (*P* = 0.002) (Table 2). Suicidal ideation increased significantly among women, younger adults (aged <39 years), those with higher educational attainment and those without pre-existing mental health conditions (*P* = 0.028, *P* = 0.048, *P* = 0.003 and *P* = 0.044, respectively). The adjusted model 1 showed that younger age (adjusted odds ratio 1.54, 95% CI 1.07–2.22, *P* = 0.021), loneliness at time point 2 (adjusted odds ratio 1.52, 95% CI 1.02–2.26, *P* = 0.041) and suicidal ideation at time point 2 (adjusted odds ratio 15.17, 95% CI 9.95–23.13, *P* < 0.001) were significantly associated with suicidal ideation at time point 3 (Table 3). The fully adjusted model 2 showed that younger age (adjusted odds ratio 1.57, 95% CI 1.09–2.28, *P* = 0.017), pre-existing mental health conditions at time point 1 (adjusted odds ratio 2.17, 95% CI 1.28–3.67, *P* = 0.004) and suicidal ideation at time point 2 (adjusted odds ratio 15.40, 95% CI 10.06–23.58, *P* < 0.001) were significantly associated with suicidal ideation at time point 3 (Table 3).

**Table 2:** The prevalence of loneliness and suicidal ideation of full-time employees at time points 2 and 3 in the total and subgroups stratified by demographic characteristics, during the COVID-19 pandemic in Japan (*N* = 875)

		Loneliness				Suicidal ideation			
		Time point 2 (May 2020),	Time point 3 (August 2020),	*P*-value for differencebetween time points 2 and 3 (McNemer)	McNemar odds ratio (95% CI)	Time point 2 (May 2020),	Time point 3 (August 2020),	*P*-value for difference between time points 2 and 3 (McNemer)	McNemar odds ratio (95% CI)
	*N*	*n* (%)	*n* (%)			*n* (%)	*n* (%)		
All	875	372 (42.5)	416 (47.5)	0.002*	1.60 (1.19–2.18)	218 (24.9)	251 (28.7)	0.008*	1.59 (1.13–2.26)
Gender									
Male	463	188 (40.6)	211 (45.6)	0.025*	1.62 (1.06–2.51)	119 (25.7)	134 (28.9)	0.129	1.43 (0.91–2.27)
Female	412	184 (44.7)	205 (49.8)	0.038*	1.58 (1.03–2.47)	99 (24.0)	117 (28.4)	0.028*	1.86 (1.07–3.32)
Age									
>40 years	492	197 (40.0)	219 (44.5)	0.039*	1.54 (1.02–2.34)	98 (19.9)	112 (22.8)	0.099	1.58 (0.93–2.76)
≤39 years	383	175 (45.7)	197 (51.4)	0.024*	1.69 (1.07–2.70)	120 (31.3)	139 (36.3)	0.048*	1.59 (1.01–2.56)
Education^a^									
Low	405	181 (44.7)	201 (49.6)	0.043*	1.59 (1.02–2.52)	106 (26.2)	111 (27.4)	0.614	1.17 (0.69–2.00)
High	470	191 (40.6)	215 (45.7)	0.023*	1.62 (1.07–2.47)	112 (23.8)	140 (29.8)	0.003*	2.04 (1.26–3.36)
Occupation type									
Managerial/non-manual	648	281 (43.4)	309 (47.7)	0.025*	1.48 (1.05–2.09)	162 (25.0)	182 (28.1)	0.060	1.49 (0.99–2.27)
Manual	227	91 (40.1)	107 (47.1)	0.024*	2.14 (1.10–4.37)	56 (24.7)	69 (30.4)	0.067	1.87 (0.96–3.76)
Pre-existing mental health condition in March 2020 (time point 1)									
No	771	308 (39.9)	345 (44.7)	0.005*	1.59 (1.15–2.21)	180 (23.3)	203 (26.3)	0.044*	1.48 (1.01–2.18)
Yes	104	64 (61.5)	71 (68.3)	0.248	1.70 (0.74–4.15)	38 (36.5)	48 (46.2)	0.078	2.25 (0.93–5.98)

a. High educational attainment was indicated as an undergraduate degree and beyond.

* *P* < 0.05.

**Table 3:** Factors associated with suicidal ideation in August 2020 (time point 3), in full-time employees during the COVID-19 pandemic in Japan (*N* = 875)

	Crude			Adjusted (model 1)^a^			Adjusted (model 2)^b^		
	Odds ratio	95% CI	*P*-value	Odds ratio	95% CI	*P*-value	Odds ratio	95% CI	*P*-value
Gender									
Male	1.00			1.00			1.00		
Female	0.97	0.73−1.31	0.859	1.14	0.78−1.67	0.490	1.12	0.77−1.65	0.552
Age									
<40 years	1.00			1.00			1.00		
≤39 years	1.93	1.44−2.60	<0.001*	1.54	1.07−2.22	0.021*	1.57	1.09−2.28	0.017*
Education^c^									
Low	1.00			1.00			1.00		
High	1.12	0.84−1.51	0.438	1.40	0.94−2.08	0.099	1.39	0.93−2.07	0.107
Occupation type									
Managerial/non-manual	1.00			1.00			1.00		
Manual	1.12	0.80−1.56	0.508	1.33	0.86−2.05	0.208	1.35	0.87−2.09	0.185
Suicidal ideation in May 2020 (time point 2)									
No	1.00			1.00			1.00		
Yes	18.46	12.66−26.92	<0.001*	15.17	9.95−23.13	<0.001*	15.40	10.06−23.58	<0.001*
Loneliness in May 2020 (time point 2)									
No	1.00			1.00			1.00		
Yes	4.17	3.05−5.69	<0.001*	1.52	1.02−2.26	0.041*	1.42	0.95−2.13	0.088
Pre-existing mental health condition in March (time point 1)									
No	1.00			−			1.00		
Yes	2.40	1.58−3.64	<0.001*	−			2.17	1.28−3.67	0.004*

a. Adjusted for gender, age, education, occupation type, and loneliness and suicidal ideation at time point 2.

b. Adjusted for gender, age, education, occupation type, and loneliness and suicidal ideation at time point 2, and pre-existing mental health condition at time point 1.

c. High educational attainment was indicated as an undergraduate degree and beyond.

* *P* < 0.05.

## Discussion

Suicidal ideation significantly increased between time point 2 (May 2020) and time point 3 (August 2020) in the total analytic sample of Japanese employees (*N* = 875). Factors significantly associated with suicidal ideation were younger age (aged <39 years), suicidal ideation at time point 2 and pre-existing mental health conditions. Loneliness at time point 2 also showed significant association, without adjusting for pre-existing mental health conditions.

Echoing the study findings by O'Connor et al,^2^ the younger population in both Japan and the UK may be at greater risk of suicidal ideation during the pandemic. In fact, since July 2020, the number of suicides in Japan each month has increased among young people and women compared with that of the same month in 2019.^6^ In more detail, monthly suicide rates in Japan declined by 14% during the first 5 months of the pandemic (February to June 2020), but monthly suicide rates increased by 16% during the second wave (July to October 2020), with a larger increase among women (37%) and children and adolescents (49%).^7^ In contrast, there was no significant association with suicidal ideation in gender among full-time employees in this study, whereas suicidal ideation longitudinally increased among women. The literature^8,9^ reported that women who were employed part-time or unemployed may be more affected by COVID-19, but they were not part of our sample. Future monitoring may be worthwhile for the female population, who are more likely to be affected by COVID-19.

Pre-existing mental health conditions and pre-existing suicidal ideation at time point 2 increased the risk of suicidal ideation at time point 3. Pre-existing psychiatric disorders are suggested as one of the risk factors for suicide during the COVID-19 pandemic.^10^ A Chinese cross-sectional study reported that psychiatric patients showed higher suicidal ideation and poorer mental health than healthy controls during early lockdown in the COVID-19 pandemic.^11^ Employees with pre-existing mental health conditions may be at risk.

Loneliness may be one of the key factors in identifying the risk of, and preventing, suicide.^12^ As almost all groups showed an increase in loneliness between time points 2 and 3 (Table 2), less social interaction (i.e. social disconnectedness) may universally be taken into consideration in the prolonged pandemic.^10^

This study had several limitations. We did not measure these variables at time point 1 (baseline), so we could not compare these with early stages of the COVID-19 pandemic. This study has limited generalisability because it was a survey of full-time employees, leading to an underestimation of the risk of suicidal ideation and loneliness by excluding people who were unemployed or who had a precarious job. The generalisability may also be limited by the online recruitment procedure and sampling bias resulting from differences in drop-out rates related to gender, age, occupation and pre-existing mental health conditions. The suicidal ideation of this study was more passive than in previous study the previous study by O'Connor et al,^2^ which collected data on the frequency of active suicidal ideation, attempts and self-harm attempts (‘How many times in the past week have you thought of taking your life?’). Such a difference may cause overestimation of the frequency of suicidal ideation in this study.

Future studies are needed for practical public health measures to reduce the risk of suicide in the COVID-19 pandemic, investigating both individual risk (e.g. illness) and social risk (e.g. finance, isolation).^13^

In summary, the results suggest that suicidal ideation of employees has increased from May to August 2020 during the ongoing COVID-19 outbreaks, parallel to the observed increased suicide cases in Japan. Being young, experiencing loneliness, having a pre-existing mental health condition and prior suicidal ideation were suggested to be a risk factor of suicidal ideation. National and community support is needed to target people who are likely to be left behind.^14,15^

## Data Availability

The data that support the findings of this study are available from the corresponding author, N.K., upon reasonable request.

## Supplementary material

For supplementary material accompanying this paper visit http://dx.doi.org/10.1192/bjo.2021.1035. click here to view supplementary material

## References

[1] John A, Pirkis J, Gunnell D, Appleby L, Morrissey J. Trends in suicide during the covid-19 pandemic. BMJ 2020; 371(m4352): m4352. 33184048 10.1136/bmj.m4352

[2] O'Connor RC, Wetherall K, Cleare S, McClelland AJ, Melson CL, Niedzwiedz RE, Mental health and wellbeing during the COVID-19 pandemic: longitudinal analyses of adults in the UK COVID-19 Mental Health & Wellbeing Study. Br J Psychiatry 2021; 218(6): 326–33. 10.1192/bjp.2020.212 PMC7684009 33081860

[3] Czeisler MÉ, Lane RI, Petrosky E, Mental health, substance use, and suicidal ideation during the COVID-19 pandemic - United States, June 24–30, 2020. Morb Mortal Wkly Rep 2020; 69(32): 1049–57. 10.15585/mmwr.mm6932a1 PMC7440121 32790653

[4] Yamamoto T, Uchiumi C, Suzuki N, Yoshimoto J, Murillo-Rodriguez E. The psychological impact of ‘mild lockdown’ in Japan during the COVID-19 pandemic: a nationwide survey under a declared state of emergency. Int J Environ Res Public Health 2020; 17(24): 9382. 10.3390/ijerph17249382 PMC7765307 33333893

[5] Sasaki N, Kuroda R, Tsuno K, Kawakami N. Workplace responses to COVID-19 associated with mental health and work performance of employees in Japan. J Occup Health 2020; 62 (1): e12134. 32529654 10.1002/1348-9585.12134 PMC7289653

[6] National Police Agency. *The Suicide Situation in 2020*. National Police Agency, 2020 (https://www.npa.go.jp/publications/statistics/safetylife/jisatsu.html).

[7] Tanaka T, Okamoto S. Increase in suicide following an initial decline during the COVID-19 pandemic in Japan. Nat Hum Behav 2021; 5(2): 229–38. 33452498 10.1038/s41562-020-01042-z

[8] Matilla-Santander N, Ahonen E, Albin M, Albin S, Baron M, Bolíbar K, COVID-19 and precarious employment: consequences of the evolving crisis. Int J Health Serv 2021; 51(2): 226–8. 33430684 10.1177/0020731420986694 PMC8114423

[9] Ruffolo M, Price D, Schoultz M, Leung J, Bonsaksen T, Thygesen H, Employment uncertainty and mental health during the COVID-19 pandemic initial social distancing implementation: a cross-national study. Glob Soc Welf [Epub ahead of print] 7 Jan 2021. Available from: 10.1007/s40609-020-00201-4. PMC7788173 33432284

[10] Raj S, Ghosh D, Singh T, Verma SK, Arya YK. Theoretical mapping of suicidal risk factors during the COVID-19 pandemic: a mini-review. Front Psychiatry 2020; 11: 589614. 33551864 10.3389/fpsyt.2020.589614 PMC7862110

[11] Hao F, Tan W, Jiang L, Do psychiatric patients experience more psychiatric symptoms during COVID-19 pandemic and lockdown? A case-control study with service and research implications for immunopsychiatry. Brain Behav Immun 2020; 87: 100–6. 32353518 10.1016/j.bbi.2020.04.069 PMC7184991

[12] Stickley A, Koyanagi A. Loneliness, common mental disorders and suicidal behavior: findings from a general population survey. J Affect Disord 2016; 197: 81–7. 26971125 10.1016/j.jad.2016.02.054

[13] Gunnell D, Appleby L, Arensman E, Hawton K, John A, Kapur N, Suicide risk and prevention during the COVID-19 pandemic. Lancet Psychiatry 2020; 7(6): 468–71. 32330430 10.1016/S2215-0366(20)30171-1 PMC7173821

[14] United Nations. Policy Brief: COVID-19 and the Need for Action on Mental Health. United Nations, 2020 (https://www.un.org/sites/un2.un.org/files/un_policy_brief-covid_and_mental_health_final.pdf).

[15] Sher L. The impact of the COVID-19 pandemic on suicide rates. QJM 2020; 113(10): 707–12. 32539153 10.1093/qjmed/hcaa202 PMC7313777

