# Supplementary material for: Increased suicidal ideation in the COVID-19 pandemic: an employee cohort in Japan
Source: BJPsych Open. 2021 Oct 29;7(6):e199. doi: 10.1192/bjo.2021.1035 (PMC8564023; doi:10.1192/bjo.2021.1035)
Supplement: Supplementary file 1 [file bjosup.zip › S2056472421010358sup001.docx]

**Supplementary table 1. The difference of characteristics at T1 (March 2020) between participants in the analytic sample (N=875) and those who dropped out or were excluded due to unemployment (N=573).**

|  | Analytic sample (N=875) | | Dropped out (N=573)† | | P for difference§ |
| --- | --- | --- | --- | --- | --- |
|  | N (%) | Mean (SD) | N (%) | Mean (SD) |  |
| Gender |  |  |  |  |  |
| Male | 463 (52.9) |  | 261 (45.5) |  | 0.006* |
| Female | 412 (47.1) |  | 312 (54.5) |  |  |
| Age |  | 41.74 (10.4) |  | 40.36 (10.8) |  |
| Over 40 years old | 492 (56.2) |  | 280 (48.9) |  | 0.006* |
| Under 39 years old | 383 (43.8) |  | 293 (51.1) |  |  |
| Educational attainment§§ |  |  |  |  |  |
| Low (< 16 years) | 405 (46.3) |  | 202 (35.3) |  | 0.162 |
| High | 470 (53.7) |  | 198 (34.6) |  |  |
| Missing | - |  | 173 (30.2) |  |  |
| Occupational type (2019) |  |  |  |  |  |
| Managers/non-manual | 648 (74.1) |  | 370 (64.6) |  | 0.010* |
| Manual | 227 (25.9) |  | 176 (30.7) |  |  |
| Missing (unemployed) | - |  | 27 (4.7) |  |  |
| Pre-existing mental health condition |  |  |  |  |  |
| No | 771 (88.1) |  | 500 (87.3) |  | 0.039* |
| Yes | 104 (11.9) |  | 46 (8.0) |  |  |
| Missing | - |  | 27 (4.7) |  |  |

SD: standard deviation.

† Including 546 participants who dropped out at T2 or T3 and 27 participants at T1 who were excluded from the analysis due to unemployment.

§ The comparison was made excluding participants with missing responses. P for difference was calculated by t-test or chi square test.

§§Educational attainment was measured at T2.
