# Supplementary material for: Increased suicidal ideation in the COVID-19 pandemic: an employee cohort in Japan
Source: BJPsych Open. 2021 Oct 29;7(6):e199. doi: 10.1192/bjo.2021.1035 (PMC8564023; doi:10.1192/bjo.2021.1035)
Supplement: Supplementary file 1 [file bjosup.zip › S2056472421010358sup002.pptx]

## Slide 1
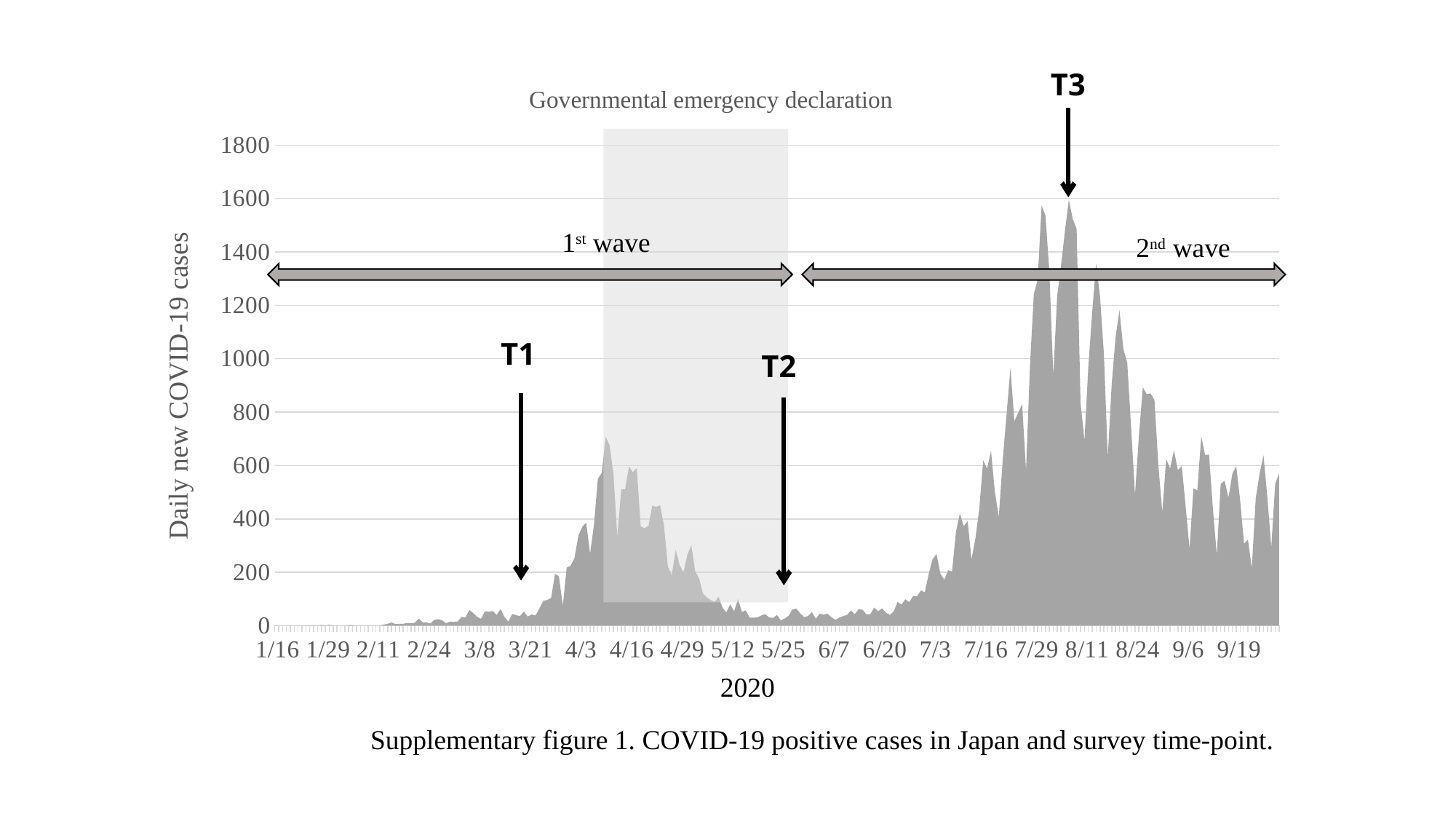

T3
Governmental emergency declaration
### Chart
| Category | |
|---|---|
| 1/16 | 1.0 |
| 1/17 | 0.0 |
| 1/18 | 0.0 |
| 1/19 | 0.0 |
| 1/20 | 0.0 |
| 1/21 | 0.0 |
| 1/22 | 0.0 |
| 1/23 | 0.0 |
| 1/24 | 1.0 |
| 1/25 | 1.0 |
| 1/26 | 1.0 |
| 1/27 | 0.0 |
| 1/28 | 3.0 |
| 1/29 | 1.0 |
| 1/30 | 3.0 |
| 1/31 | 1.0 |
| 2/1 | 0.0 |
| 2/2 | 0.0 |
| 2/3 | 0.0 |
| 2/4 | 2.0 |
| 2/5 | 2.0 |
| 2/6 | 0.0 |
| 2/7 | 0.0 |
| 2/8 | 0.0 |
| 2/9 | 0.0 |
| 2/10 | 0.0 |
| 2/11 | 0.0 |
| 2/12 | 1.0 |
| 2/13 | 4.0 |
| 2/14 | 7.0 |
| 2/15 | 12.0 |
| 2/16 | 6.0 |
| 2/17 | 7.0 |
| 2/18 | 7.0 |
| 2/19 | 10.0 |
| 2/20 | 9.0 |
| 2/21 | 11.0 |
| 2/22 | 27.0 |
| 2/23 | 12.0 |
| 2/24 | 12.0 |
| 2/25 | 8.0 |
| 2/26 | 22.0 |
| 2/27 | 24.0 |
| 2/28 | 20.0 |
| 2/29 | 9.0 |
| 3/1 | 15.0 |
| 3/2 | 14.0 |
| 3/3 | 16.0 |
| 3/4 | 33.0 |
| 3/5 | 31.0 |
| 3/6 | 59.0 |
| 3/7 | 47.0 |
| 3/8 | 33.0 |
| 3/9 | 26.0 |
| 3/10 | 54.0 |
| 3/11 | 52.0 |
| 3/12 | 55.0 |
| 3/13 | 40.0 |
| 3/14 | 62.0 |
| 3/15 | 33.0 |
| 3/16 | 15.0 |
| 3/17 | 44.0 |
| 3/18 | 39.0 |
| 3/19 | 36.0 |
| 3/20 | 53.0 |
| 3/21 | 34.0 |
| 3/22 | 42.0 |
| 3/23 | 38.0 |
| 3/24 | 65.0 |
| 3/25 | 93.0 |
| 3/26 | 96.0 |
| 3/27 | 104.0 |
| 3/28 | 194.0 |
| 3/29 | 185.0 |
| 3/30 | 74.0 |
| 3/31 | 218.0 |
| 4/1 | 224.0 |
| 4/2 | 253.0 |
| 4/3 | 337.0 |
| 4/4 | 370.0 |
| 4/5 | 386.0 |
| 4/6 | 270.0 |
| 4/7 | 377.0 |
| 4/8 | 550.0 |
| 4/9 | 572.0 |
| 4/10 | 708.0 |
| 4/11 | 676.0 |
| 4/12 | 571.0 |
| 4/13 | 333.0 |
| 4/14 | 511.0 |
| 4/15 | 511.0 |
| 4/16 | 596.0 |
| 4/17 | 575.0 |
| 4/18 | 590.0 |
| 4/19 | 372.0 |
| 4/20 | 365.0 |
| 4/21 | 374.0 |
| 4/22 | 450.0 |
| 4/23 | 445.0 |
| 4/24 | 451.0 |
| 4/25 | 374.0 |
| 4/26 | 221.0 |
| 4/27 | 189.0 |
| 4/28 | 284.0 |
| 4/29 | 227.0 |
| 4/30 | 199.0 |
| 5/1 | 266.0 |
| 5/2 | 302.0 |
| 5/3 | 204.0 |
| 5/4 | 177.0 |
| 5/5 | 120.0 |
| 5/6 | 106.0 |
| 5/7 | 96.0 |
| 5/8 | 89.0 |
| 5/9 | 108.0 |
| 5/10 | 68.0 |
| 5/11 | 50.0 |
| 5/12 | 80.0 |
| 5/13 | 54.0 |
| 5/14 | 100.0 |
| 5/15 | 52.0 |
| 5/16 | 57.0 |
| 5/17 | 30.0 |
| 5/18 | 30.0 |
| 5/19 | 31.0 |
| 5/20 | 38.0 |
| 5/21 | 43.0 |
| 5/22 | 31.0 |
| 5/23 | 29.0 |
| 5/24 | 40.0 |
| 5/25 | 20.0 |
| 5/26 | 27.0 |
| 5/27 | 37.0 |
| 5/28 | 61.0 |
| 5/29 | 64.0 |
| 5/30 | 46.0 |
| 5/31 | 32.0 |
| 6/1 | 36.0 |
| 6/2 | 51.0 |
| 6/3 | 26.0 |
| 6/4 | 45.0 |
| 6/5 | 41.0 |
| 6/6 | 45.0 |
| 6/7 | 32.0 |
| 6/8 | 22.0 |
| 6/9 | 30.0 |
| 6/10 | 36.0 |
| 6/11 | 40.0 |
| 6/12 | 57.0 |
| 6/13 | 43.0 |
| 6/14 | 62.0 |
| 6/15 | 60.0 |
| 6/16 | 42.0 |
| 6/17 | 43.0 |
| 6/18 | 68.0 |
| 6/19 | 54.0 |
| 6/20 | 65.0 |
| 6/21 | 49.0 |
| 6/22 | 40.0 |
| 6/23 | 53.0 |
| 6/24 | 89.0 |
| 6/25 | 79.0 |
| 6/26 | 99.0 |
| 6/27 | 88.0 |
| 6/28 | 111.0 |
| 6/29 | 110.0 |
| 6/30 | 132.0 |
| 7/1 | 125.0 |
| 7/2 | 194.0 |
| 7/3 | 249.0 |
| 7/4 | 268.0 |
| 7/5 | 195.0 |
| 7/6 | 172.0 |
| 7/7 | 208.0 |
| 7/8 | 203.0 |
| 7/9 | 352.0 |
| 7/10 | 420.0 |
| 7/11 | 373.0 |
| 7/12 | 391.0 |
| 7/13 | 248.0 |
| 7/14 | 327.0 |
| 7/15 | 440.0 |
| 7/16 | 619.0 |
| 7/17 | 588.0 |
| 7/18 | 655.0 |
| 7/19 | 501.0 |
| 7/20 | 407.0 |
| 7/21 | 618.0 |
| 7/22 | 792.0 |
| 7/23 | 966.0 |
| 7/24 | 766.0 |
| 7/25 | 798.0 |
| 7/26 | 830.0 |
| 7/27 | 581.0 |
| 7/28 | 968.0 |
| 7/29 | 1242.0 |
| 7/30 | 1297.0 |
| 7/31 | 1574.0 |
| 8/1 | 1535.0 |
| 8/2 | 1324.0 |
| 8/3 | 937.0 |
| 8/4 | 1234.0 |
| 8/5 | 1350.0 |
| 8/6 | 1479.0 |
| 8/7 | 1595.0 |
| 8/8 | 1523.0 |
| 8/9 | 1486.0 |
| 8/10 | 836.0 |
| 8/11 | 693.0 |
| 8/12 | 969.0 |
| 8/13 | 1176.0 |
| 8/14 | 1356.0 |
| 8/15 | 1234.0 |
| 8/16 | 1017.0 |
| 8/17 | 630.0 |
| 8/18 | 904.0 |
| 8/19 | 1080.0 |
| 8/20 | 1182.0 |
| 8/21 | 1036.0 |
| 8/22 | 985.0 |
| 8/23 | 739.0 |
| 8/24 | 491.0 |
| 8/25 | 712.0 |
| 8/26 | 893.0 |
| 8/27 | 867.0 |
| 8/28 | 870.0 |
| 8/29 | 844.0 |
| 8/30 | 598.0 |
| 8/31 | 425.0 |
| 9/1 | 624.0 |
| 9/2 | 589.0 |
| 9/3 | 656.0 |
| 9/4 | 583.0 |
| 9/5 | 598.0 |
| 9/6 | 447.0 |
| 9/7 | 288.0 |
| 9/8 | 514.0 |
| 9/9 | 507.0 |
| 9/10 | 709.0 |
| 9/11 | 639.0 |
| 9/12 | 641.0 |
| 9/13 | 439.0 |
| 9/14 | 265.0 |
| 9/15 | 531.0 |
| 9/16 | 543.0 |
| 9/17 | 480.0 |
| 9/18 | 569.0 |
| 9/19 | 597.0 |
| 9/20 | 469.0 |
| 9/21 | 307.0 |
| 9/22 | 322.0 |
| 9/23 | 216.0 |
| 9/24 | 477.0 |
| 9/25 | 570.0 |
| 9/26 | 638.0 |
| 9/27 | 478.0 |
| 9/28 | 294.0 |
| 9/29 | 531.0 |
| 9/30 | 570.0 |
1st wave
2nd wave
T1
T2
Daily new COVID-19 cases
2020
Supplementary figure 1. COVID-19 positive cases in Japan and survey time-point.
