# Supplementary material for: Increased suicidal ideation in the COVID-19 pandemic: an employee cohort in Japan
Source: BJPsych Open. 2021 Oct 29;7(6):e199. doi: 10.1192/bjo.2021.1035 (PMC8564023; doi:10.1192/bjo.2021.1035)
Supplement: Supplementary file 1 [file bjosup.zip › S2056472421010358sup003.pptx]

## Slide 1
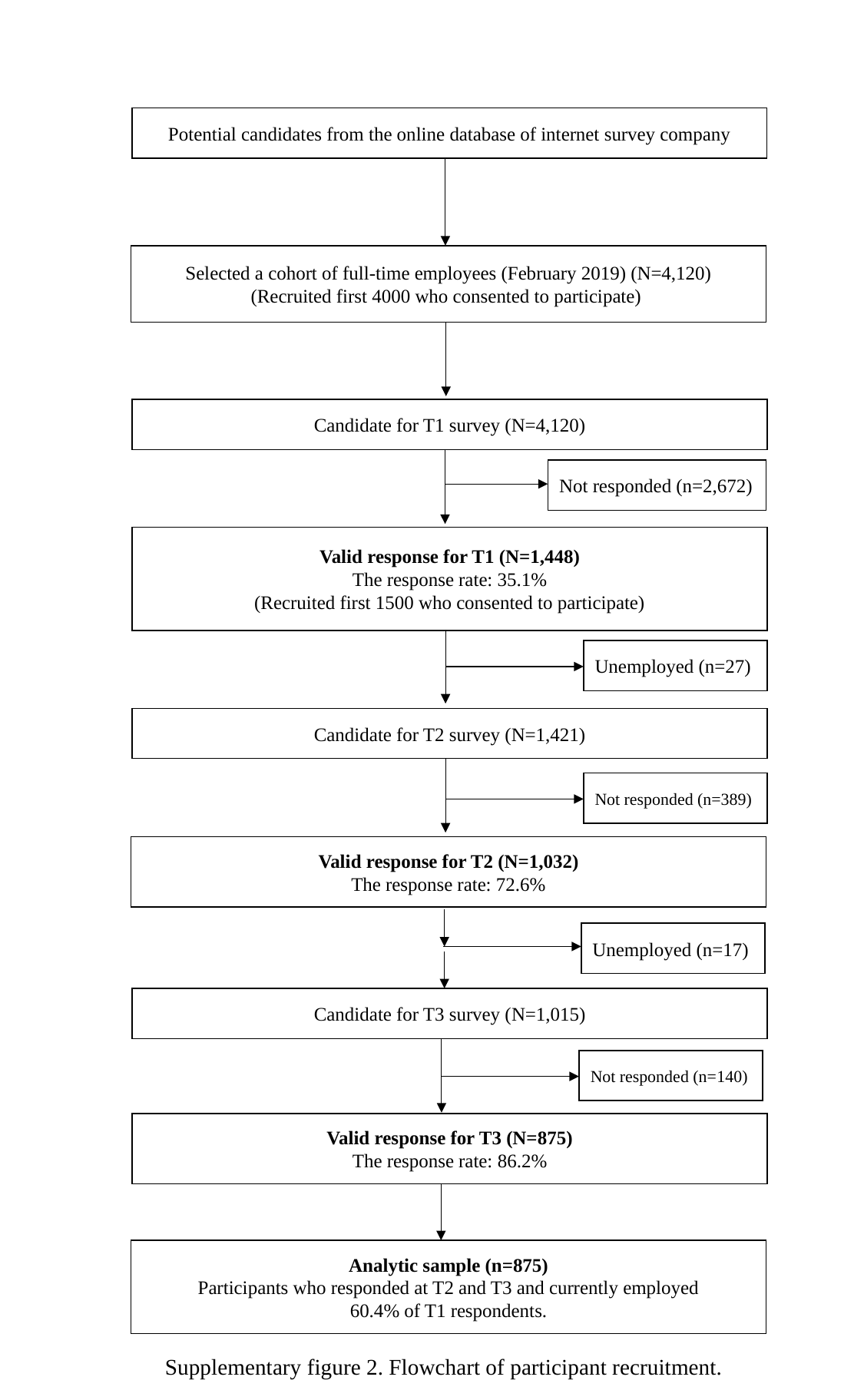

Potential candidates from the online database of internet survey company
Selected a cohort of full-time employees (February 2019) (N=4,120)
(Recruited first 4000 who consented to participate)
Candidate for T1 survey (N=4,120)
Not responded (n=2,672)
Valid response for T1 (N=1,448)
The response rate: 35.1%
(Recruited first 1500 who consented to participate)
Unemployed (n=27)
Candidate for T2 survey (N=1,421)
Not responded (n=389)
Valid response for T2 (N=1,032)
The response rate: 72.6%
Unemployed (n=17)
Candidate for T3 survey (N=1,015)
Not responded (n=140)
Valid response for T3 (N=875)
The response rate: 86.2%
Analytic sample (n=875)
Participants who responded at T2 and T3 and currently employed
60.4% of T1 respondents.
Supplementary figure 2. Flowchart of participant recruitment.
